# A Deep Learning Computer-Aided Diagnosis Approach for Breast Cancer

**DOI:** 10.3390/bioengineering9080391

**Published:** 2022-08-15

**Authors:** Ahmed M. Zaalouk, Gamal A. Ebrahim, Hoda K. Mohamed, Hoda Mamdouh Hassan, Mohamed M. A. Zaalouk

**Affiliations:** 1 Computer and Systems Engineering Department, Faculty of Engineering, Ain Shams University, Cairo 11517, Egypt; 2 School of Computing, Coventry University—Egypt Branch, Hosted at the Knowledge Hub Universities, Cairo, Egypt; 3 Department of Information Sciences and Technology, College of Engineering and Computing, George Mason University, Fairfax, VA 22030, USA; 4 Faculty of Medicine, Ain Shams University, Cairo 11591, Egypt

**Keywords:** BreakHis, breast cancer, computer-aided diagnosis, deep learning, histopathological images

## Abstract

Breast cancer is a gigantic burden on humanity, causing the loss of enormous numbers of lives and amounts of money. It is the world’s leading type of cancer among women and a leading cause of mortality and morbidity. The histopathological examination of breast tissue biopsies is the gold standard for diagnosis. In this paper, a computer-aided diagnosis (CAD) system based on deep learning is developed to ease the pathologist’s mission. For this target, five pre-trained convolutional neural network (CNN) models are analyzed and tested—Xception, DenseNet201, InceptionResNetV2, VGG19, and ResNet152—with the help of data augmentation techniques, and a new approach is introduced for transfer learning. These models are trained and tested with histopathological images obtained from the BreakHis dataset. Multiple experiments are performed to analyze the performance of these models through carrying out magnification-dependent and magnification-independent binary and eight-class classifications. The Xception model has shown promising performance through achieving the highest classification accuracies for all the experiments. It has achieved a range of classification accuracies from 93.32% to 98.99% for magnification-independent experiments and from 90.22% to 100% for magnification-dependent experiments.

## 1. Introduction

Breast cancer claims the lives of 40,000 women in the United States each year [1]. Twelve percent of women are diagnosed annually with breast cancer. It usually affects women over the age of 40 [2]. In recent years, the healthcare cost of a wide spectrum of diseases has crippled the economies of the world nations. A disease such as cancer can be extremely costly in terms of lives, quality of life, and money. Breast cancer occupies a unique place in this category of diseases, as breast cancer is the world’s leading type of cancer in female patients. In 2020, 2.3 million women were diagnosed with this disease, and it caused 685,000 fatalities. Moreover, 7.8 million women were diagnosed with this disease in the previous five years as of the end of 2020, making it the most common cancer in the world [3]. The lost disability-adjusted year rate in women is the highest in breast cancer compared to any other disease. The early identification of pre-cancerous and cancerous cases proved to be extremely effective in providing higher outcomes of cure. This was concluded from the fact that the developed countries achieved better survival rates in 1970s after early detection and intervention programs [4]. There are several tools that can be used to screen and diagnose breast cancer. These tools fall into three main categories: clinical examination, imaging techniques, and histopathological examination of tissue biopsies [5]. The gold standard for the diagnosis is tissue biopsy examination [6]. Thus, myriads of samples are produced annually, which can be considered as a huge burden on the pathologists to examine and accurately identify the diseases in it. The examination is conducted by placing a breast tissue stained by special stains, most commonly by hematoxylin and eosin, in a glass slide and examined under a microscope. Different magnification lenses are used to examine the slide. If the pathologist detects cellular atypia (general criteria of malignancy) or a tissue abnormality (e.g., ductal invasion), then the diagnosis is concluded, and the subtype of the cancer is determined [7].

In order to aid the pathologists in diagnostic process, a computer-aided diagnosis (CAD) system can be produced to reduce the errors, effort, time, and cost in the process. The system is just a helping tool and the pathologist’s judgment is irreplaceable for the diagnostic process. One way to develop a CAD system is to rely on the extraction of handcrafted features from the histopathological images, mainly to train traditional machine learning models and predict unseen inputs later. However, this approach is not popular, as it requires extensive prior-domain knowledge for the extraction of the handcrafted features. Additionally, the generated classification accuracy from such systems is insufficient to attain trust for diagnosis in such a field [8]. An alternative approach is to use the current advances in deep learning for the development of such systems. This approach does not require any prior domain knowledge, as the model learns intrinsic features from the input raw images. Additionally, the generated classification accuracy is very impressive if such a system is well designed. However, deep learning models require a huge number of input images to be trained effectively to have a great generalization ability on unseen data. This was a huge issue for histopathological images for breast cancer, until the release of BreakHis dataset, which contains 7909 histopathological images [9]. Still, this number of images is not enough for the development of a high accuracy image recognition system.

The advances in deep learning and image processing lead to the possibility of using BreakHis dataset for the development of a high-performance CAD system. This is attained by using pre-trained model(s) and data augmentation techniques. Hence, in this paper, a diagnostic system based on deep learning characterized by high accuracy in the diagnostic process is introduced. This system can classify breast cancer from histopathological images. It achieves the classification whether this classification is magnification dependent or magnification independent for both binary and multi-classification modes. To reach these objectives, a vast performance analysis has been performed for many well-known convolutional neural network (CNN) architectures, mainly to find robust CNN model(s) for the classification of breast cancer in histopathological images. A set of pre-trained CNN models are adopted, which are Xception, DenseNet201, InceptionResNetV2, VGG19, and ResNet152. These models are trained and tested using the BreakHis dataset. Moreover, two data augmentation techniques are implemented, which are rotation and horizontal flip. Furthermore, a new approach for transfer learning is introduced in this study, where each pre-trained model is trained twice, resulting in a two-phase training process. In the first phase of training, all the layers are frozen except for the fully connected layers. A high learning rate is used in this step of training. Meanwhile, in the second phase of training, all the layers are unfrozen to fine-tune the model. This phase aims to accomplish a remarkable increase in the performance of the model generated from the first round of training. Two different learning rates are used in the second round of training to determine the best model. In the two phases, the best performed model on the input dataset is monitored using the validation accuracy metric. If an improvement in the validation accuracy is noted, then the model weights are saved for this epoch. Hence, the output of each training phase is the best performed model on the input validation dataset in terms of validation accuracy.

The study presented in this paper is contrasted to the related ones by the fact that the adopted models perform testing on samples classified according to the variable magnification or independent of it. Additionally, it expresses the results as either binary classification or eight-class classification. On the contrary, most papers perform this testing magnification dependent or independent solely and expressing the classification results as binary or eight-class classification only. Furthermore, the single most effective model is targeted by this study to be used in all the experiments and be produced as the prime diagnostic model for the pathologist. Moreover, due to the lack of usage of several models generated in a single experiment in the prior research efforts, various models are used per experiment to assess the potential of attaining various successful models in a single experiment.

The rest of the paper is organized as follows: Section 2 presents the related work to this study, while Section 3 discusses the architectures and the features of the adopted pre-trained CNN architectures. Section 4 details the adopted dataset. Meanwhile, Section 5 presents the methodology used in this work including the data pre-processing techniques, the transfer learning strategy, the hyperparameter configurations, and the adopted performance metrics. Section 6 presents the detailed experimental results, followed by Section 7, which discusses the obtained results. Finally, Section 8 concludes the paper.

## 2. Related Work

Boumaraf et al. [8] implement a transfer learning-based CNN using ResNet-18 model. They used the BreakHis dataset, and they divided it into 80% training set and 20% testing set. The experiments carried out are magnification-dependent and magnification-independent classification for both binary and multi-class (eight classes) classifications. ResNet-18 is trained on the ImageNet database. The transfer learning strategy is to train only the last two residual blocks and freeze the rest of the blocks; consequently, it makes ResNet more domain-specific (to learn the intrinsic features of the histopathology images). Meanwhile, they have first resized the input images size to 224 × 224. Additionally, global contrast normalization (GCN) has been adopted to prevent the images from having different values of contrast. Moreover, three-fold data augmentation has been used, in which each image is transformed into three images by applying three transformation techniques, which are random horizontal flip, random vertical flip, and random rotation with 40°. The metrics used to evaluate the performance are accuracy, precision, recall, F-measure, and Matthews’ correlation coefficient. The study achieves 98.42% accuracy in magnification-independent binary classification and 92.03% in magnification-independent multi-classification. Moreover, 98.84% average accuracy in magnification-dependent binary classification and 92.15% average accuracy in magnification-dependent multi-classification are achieved. The detailed results are illustrated in Table 1.

Davoudi et al. [10] design and implement a CNN for the detection of binary classes of BreakHis dataset independent of the magnification factors. The main contribution of their study is to try to optimize the weights of the CNN using genetic algorithms (GAs) instead of the normal optimizers. The model is trained using Adam, mini-batch gradient descent, and the GA optimizers. The evaluation metrics used in this study are accuracy, recall, precision, F1-score, and execution time. They divide the BreakHis dataset as 70% training set and 30% testing set. The model achieves 69.88% accuracy with gradient descent optimizer, 85.83% accuracy with Adam optimizer and 85.49% accuracy with the GA optimizer. The model accepts the images with a size of 210 × 210 × 3. Table 1 illustrates their results.

Spanhol et al. [11] tested the use of DeCaf features. DeCaf is simply the usage of a pre-trained CNN as a feature vector with a classifier on top of it that is trained for the new classification task. In more detail, an output of a given layer of a pre-trained network is used as an input to a classifier. Logistic regression has been adopted as the classifier for this study. BVLC CaffeNet is used as the pre-trained model in their study. The study considers patch-based recognition and different configurations for it. Firstly, they test the output from three layers solely. These layers are fc6, fc7, and fc8. After that, they consider the features through combining the output from more than one layer. The classification tasks performed in this study are the binary magnification-dependent tasks. Image-level accuracy and patient-level accuracy are used as the performance metrics. Additionally, they consider the F1-score at the patient level and image level. The highest patient-level accuracy is obtained for the 200× dataset, and it is equal to 86.3 ± 3.5. Meanwhile, the highest image-level accuracy is computed for the 200× dataset, and it is equal to 84.2 ± 1.7. The detailed patient-level and image-level accuracies for each magnification factor is presented in Table 1.

Bardou et al. [12] implement many approaches for the classification of breast cancer using the BreakHis dataset. The preformed experiments are magnification-dependent binary classification and multi-class classification. A CNN model is proposed in their study with five convolutional layers and two fully connected layers. This model has been developed with the original dataset without data augmentation in one trial and with data augmentation in another trial through applying horizontal flip and rotation with three angles: 90°, 180°, and 270°. Additionally, they test the CNN + support vector machine (SVM) configuration through using a linear support vector machine instead of the fully connected layer, CNN + classifiers, which is simply the usage of CNN to extract features and then classifying them through random forests, radial basis support vector machine, linear support vector machine, and K-nearest neighbors. Another approach they follow is the usage of an ensemble model in which they use 10 models, and then the probability vector of each sample in the test set is extracted from the last fully connected layer (with softmax activation) of each model, creating 10 probability vectors. Finally, these 10 probability vectors are summed up and the maximum value is computed among them to output the predicted class. Moreover, they extract handcrafted features from the images and then they classify them with traditional classifiers such as support vector machine and CNN (the extracted handcrafted features are given as input to the CNN). Moreover, they divide the dataset into 70% training set and 30% test set. The evaluation metrics used in their study are accuracy, precision, recall, and F1-score. The highest results are achieved by the ensemble model, in which they achieve accuracy in the interval [96.15%, 98.33%] for the binary classification experiments and [83.31%, 88.23%] for the multiclassification experiments. Table 1 illustrates these results in more details.

Xiang et al. [13] uses a pre-trained Inception-V3 model for the detection of malignant and benign tumours. They carry out malignant and benign magnification-independent classification. The dataset used is BreakHis dataset. The input size for their model is 229 × 229. Data augmentation techniques are used to overcome overfitting, mainly using image flip and rotation techniques. They flip each image and rotate them around their centers with angles of 90°, 180°, and 270°. Using a data augmentation strategy, they increase the BreakHis dataset by five times. The dataset is divided into train, validation, and test sets with an approximate ratio of 3:1:1. A cross-validation training strategy is adopted. The evaluation metrics used in their study are the image-classification rate and patient-classification rate. The best results are achieved using the cross-validation training strategy on the expanded dataset with an image accuracy of 95.7% and patient accuracy of 97.2%. The detailed results are shown in Table 1.

Shallu et al. [14] conduct a study to determine whether to use transfer learning or a fully trained model to classify the histopathological images in the BreakHis dataset. Three pre-trained models for this task are used: VGG16, VGG19, and ResNet50. These models are used as feature extractors only and they have used logistic regression as a classifier. Moreover, three different training and testing splitting ratios are used to determine the effect of using different ratios on the results. The ratios used are 90–10%, 80–20%, and 70–30%. Only rotation is used for the data augmentation, where the images are rotated around their centers with three different angles: 90°, 180°, and 270°. A binary magnification-independent experiment on the BreakHis dataset is carried out in this study to create a balanced dataset for the process of fine-tuning and the full training of the models. 

**Table 1:** Performance results achieved by the related models.

Reference	Classification Type	Dataset Splitting Ratio	Accuracy (%)	Precision (%)	Recall (%)	F1-Score (%)	MCC	Patient Level Accuracy (%)	Average Precision Score (%)
Boumaraf et al. [8]	Magnification-independent binary classification	80% training set and 20% testing set	98.42	98.75	99.01	98.88	0.9619	-	-
	Magnification-independent multi-classification		92.03	91.39	90.28	90.77	0.8938	-	-
	40× binary classification		99.25	99.63	99.26	99.44	0.9829	-	-
	100× binary classification		99.04	98.99	99.66	99.33	0.9765	-	-
	200× binary classification		99.00	98.94	99.65	99.29	0.9762	-	-
	400× binary classification		98.08	98.00	99.19	98.59	0.9558	-	-
	40× multi-classification		94.49	93.81	94.78	94.15	0.9283	-	-
	100× multi-classification		93.27	92.94	91.59	92.23	0.9141	-	-
	200× multi- classification		91.29	91.18	88.28	89.47	0.8895	-	-
	400× multi- classification		89.56	87.97	87.97	87.77	0.8652	-	-
Davoudi et al. [10]	Magnification-independent binary classification with Gradient descent optimizer	70% training set and 30% testing set	69.88	84.37	55.61	67.02	-	-	-
	Magnification-independent binary classification with Adam optimizer		85.83	96.23	72.31	82.56	-	-	-
	Magnification-independent binary classification with GA optimizer		85.49	94.71	69.43	80.11	-	-	-
Spanhol et al. [11]	40× binary classification	-	84.6 ± 2.9	-	-	-	-	84.0 ± 6.9	-
	100× binary classification		84.8 ± 4.2	-	-	-	-	83.9 ± 5.9	-
	200×binary classification		84.2 ± 1.7	-	-	-	-	86.3 ± 3.5	-
	400× binary classification		81.6 ± 3.7	-	-	-	-	82.1 ± 2.4	-
Bardou et al. [12]	40× binary classification	70% training set and 30% testing set	98.33	97.80	97.57	97.68	-	-	-
	100× binary classification		97.12	98.58	96.98	97.77	-	-	-
	200× binary classification		97.85	95.61	99.28	97.41	-	-	-
	400× binary classification		96.15	97.54	96.49	97.01	-	-	-
	40× multi-classification		88.23	84.27	83.79	83.74	-	-	-
	100× multi-classification		84.64	84.29	84.48	84.31	-	-	-
	200× multi- classification		83.31	81.85	80.83	80.48	-	-	-
	400× multi- classification		83.98	80.84	81.03	80.63	-	-	-
Xiang et al. [13]	Magnification-independent binary classification with normal training strategy and original database	3:1:1-training, validation, and testing sets	92.8	-	-	-	-	93.6	-
	Magnification-independent binary classification with normal training strategy and expanded database		94.6	-	-	-	-	95.4	-
	Magnification-independent binary classification with cross validation training strategy and original database		93.2	-	-	-	-	94.1	-
	Magnification-independent binary classification with cross validation training strategy and expanded database		95.7	-	-	-	-	97.2	-
Shallu et al. [14]	Magnification-independent binary classification using fine-tuned pre-trained VGG-16 network with logistic regression classifier	90% training set and 10% testing set	92.60	93	93	93	-	-	95.95
Min Liu et al. [15]	40× binary classification	60% training set, 20% validation set and 20% testing set	98.15 ± 0.9	-	-	-	-	-	-
	100× binary classification		97.71 ± 1.9	-	-	-	-	-	-
	200× binary classification		97.96 ± 0.7	-	-	-	-	-	-
	400× binary classification		98.48 ± 1.1	-	-	-	-	-	-

The images in the malignant class (which are greater than the images of the benign class) are down-sampled to a number equal to the number of images in the benign class. To fully train the networks from scratch, the weights are initialized randomly. However, they have kept the weights of the pre-trained networks without change for the transfer-learning approach. The performance measures used are accuracy, precision, recall, F1-score, and average precision score (APS). In addition, they use receiver operating characteristics (ROC) and area under the curve (AUC) to further validate the model performance. The results show that the fined-tuned VGG-16 has the best performance with 92.60% accuracy using the 90–10% training and testing data-splitting ratio. Table 1 presents the best results obtained for VGG-16.

Liu et al. [15] implement a CNN model called the AlexNet-BC model. This model is pre-trained on the ImageNet dataset and then fine-tuned using transfer learning. Moreover, many data augmentation techniques are used to expand the dataset. Additionally, a new loss function approach is proposed and implemented. The proposed model is trained and tested using the BreakHis dataset for the four different magnification factors in binary classification mode, and then the model is further verified using UCSB and IDC datasets. They have divided the BreakHis dataset into 60% for the training set, 20% for the validation set, and 20% for the test set. One evaluation metric is used in this study, which is accuracy. They have achieved a range of accuracies in the interval [97.71 ± 1.9%, 98.48 ± 1.1%]. Since Table 1 consists of results achieved by previous studies using the BreakHis dataset, we only illustrate in Table 1 the results achieved by this study using the BreakHis dataset.

## 3. Pre-Trained CNN Architectures

In this section, the architectures and the features of the adopted pre-trained models in this study are going to be discussed.

### 3.1. VGG-19

**Figure 1:**
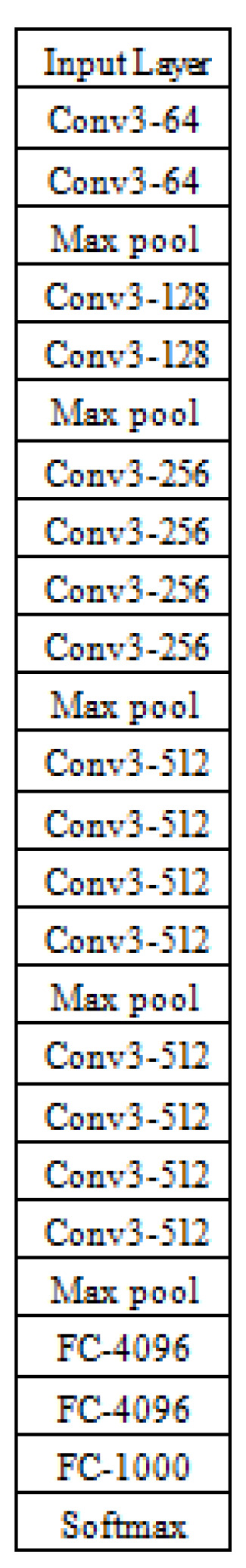
Architecture of VGG-19.

Figure 1 illustrates the architecture of VGG-19. The main idea addressed by this architecture is the possibility to increase the depth of a CNN model using too small convolutional filters of size 3 × 3 in every layer of the architecture. Five max-pooling layers exist to perform the spatial pooling process. Every max-pooling operation is performed using a 2 × 2 filter and a stride of 2. The convolution stride is always set to 1. It is clear from Figure 1 that there are 19 learnable layers, which are 16 convolutional layers and 3 fully connected layers. The numbering of filters starts at 64 and then increases by a factor of 2 along the depth of the network. The third fully connected layer has 1000 neurons as this network is designed based on the ILSVRC classification task [16].

### 3.2. ResNet-152

**Figure 2:**
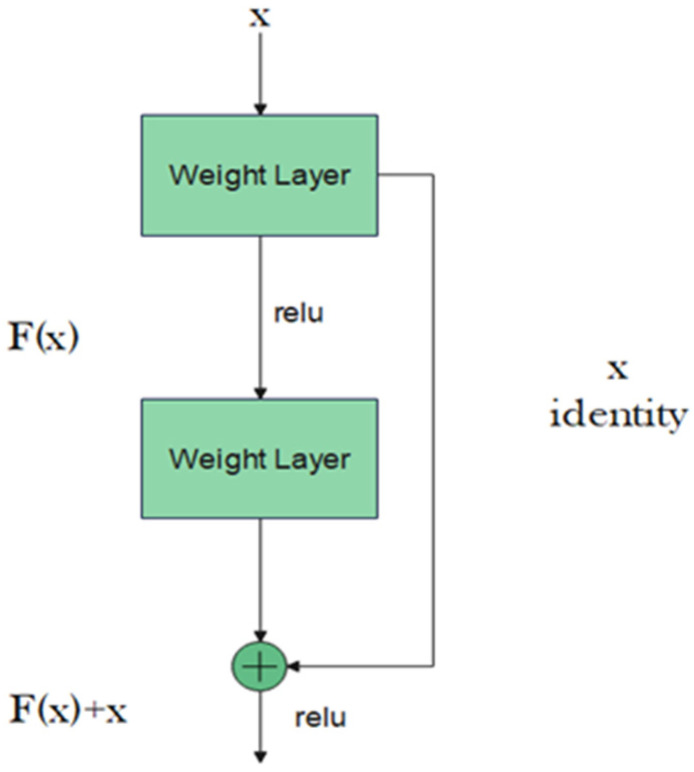
Residual block.

Residual neural network (ResNet) is another attempt to train deeper neural networks while achieving a high classification result. One could expect that a deeper network should perform at least equally to the performance achieved by its counterpart shallower network. However, this is not usually the case: the deeper the network, the more its training error increases. ResNet addresses this degradation in performance through the addition of residual blocks that employ the idea of shortcut connections as shown in Figure 2 [17].

**Figure 3:**
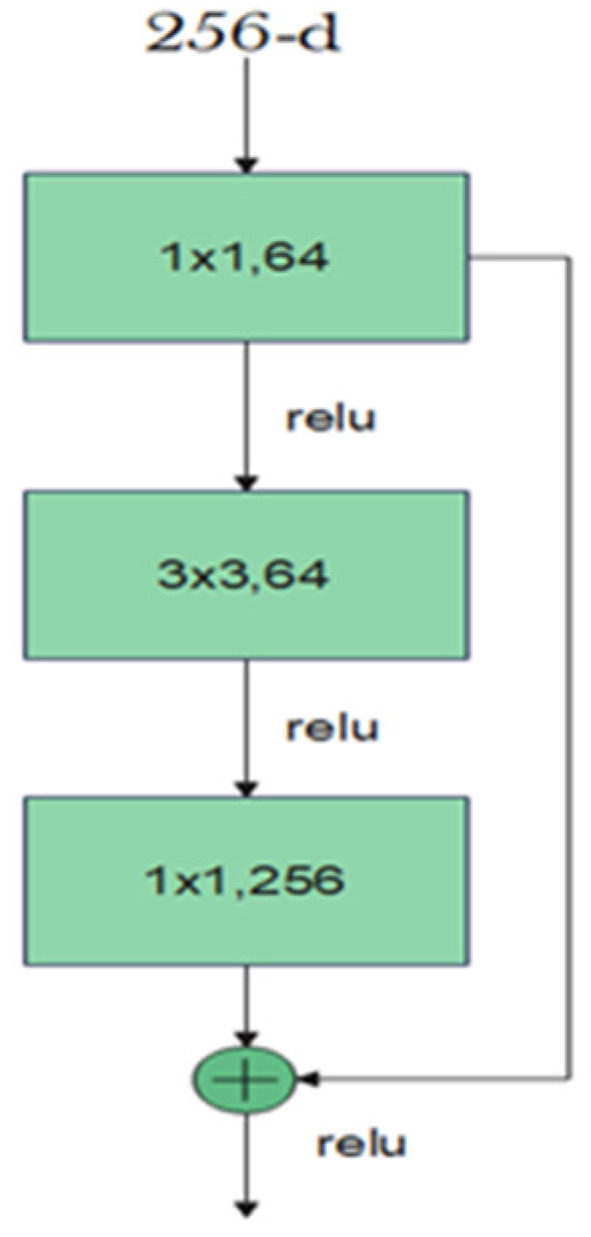
Bottleneck design of the residual block.

**Figure 4:**
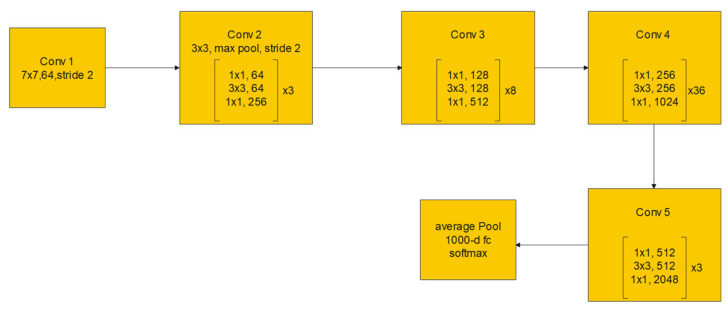
ResNet-152 architecture.

The residual block consists of two convolutional layers, in which the output of this block *F*(*x*) is added to the input *x* of this block via the shortcut connection. Simply, *F*(*x*) + *x* can be implemented easily using a feedforward neural network and shortcut connections that skip one or more stacked layers. The main purpose of the shortcut connections is that they perform identity mapping without adding any extra cost in terms of computational complexity and extra parameters. With the help of these residual blocks, a deeper model should have a training error no larger than that achieved through its shallower counterpart network. One variant of the residual block is introduced for deeper networks. This variant is a bottleneck design of the normal residual block. It consists of three convolutional layers: 1 × 1, 3 × 3, and 1 × 1 convolutions. The aim of this new variant is to decrease the number of feature maps for computational efficiency. Figure 3 represents this bottleneck design. ResNet-152 uses several three-layer blocks and its architecture is illustrated in Figure 4 [17].

### 3.3. InceptionResNetV2

**Figure 5:**
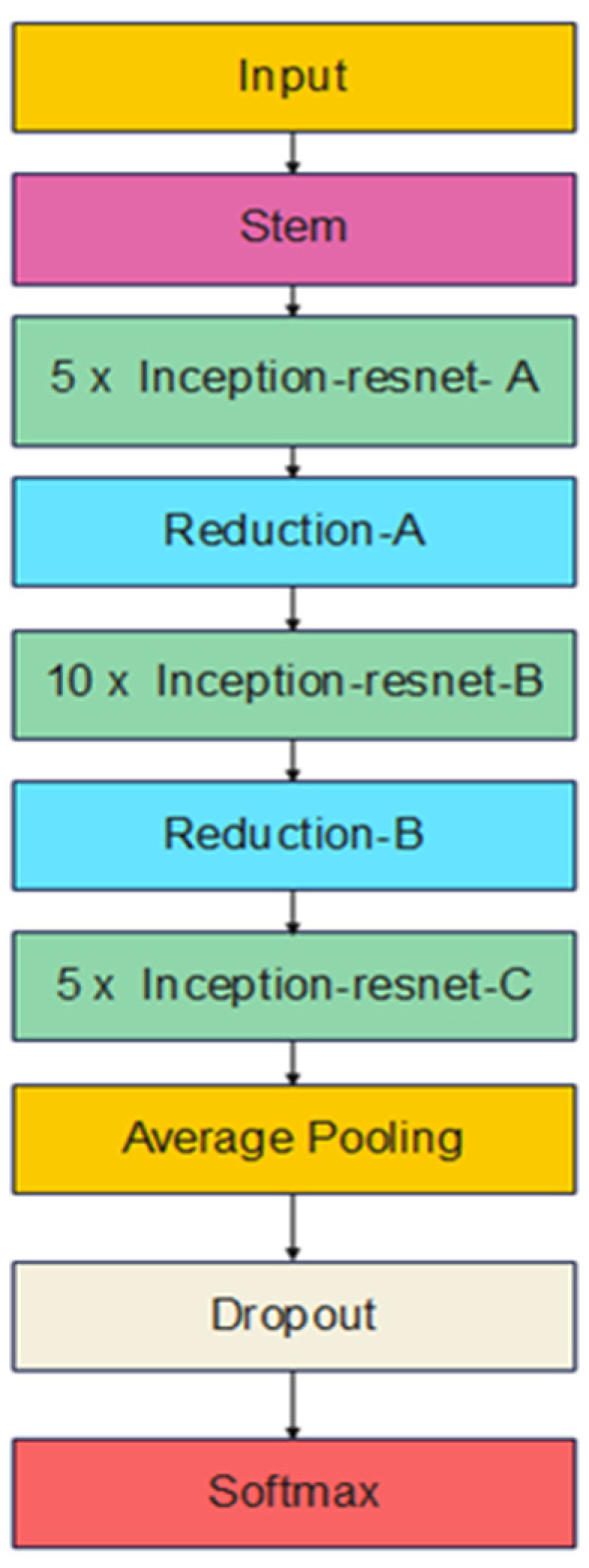
InceptionResNetV2 architecture.

Szegedy et al. [18] try to answer the question of whether to incorporate the residual connections with the inception architecture, mainly to improve the training process of the Inception network and to improve its classification performance. InceptionResNetV2 is one of the solutions to this question. As Inception architecture is very deep, residual connections replaces the filter concatenation stage of the Inception architecture. The results show that a significant improvement in the classification performance is achieved by this new hybrid model. Figure 5 illustrates an abstract view of the InceptionResNetV2 architecture. A detailed view for every block of Figure 5 is available in [18].

### 3.4. DenseNet-201

**Figure 6:**
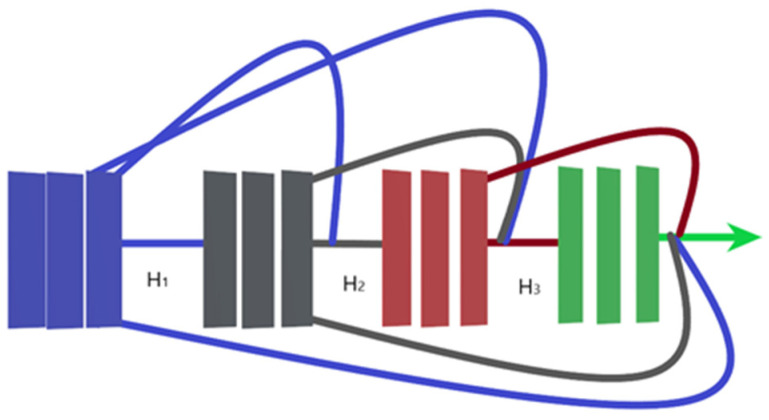
Three-layers dense block.

**Figure 7:**
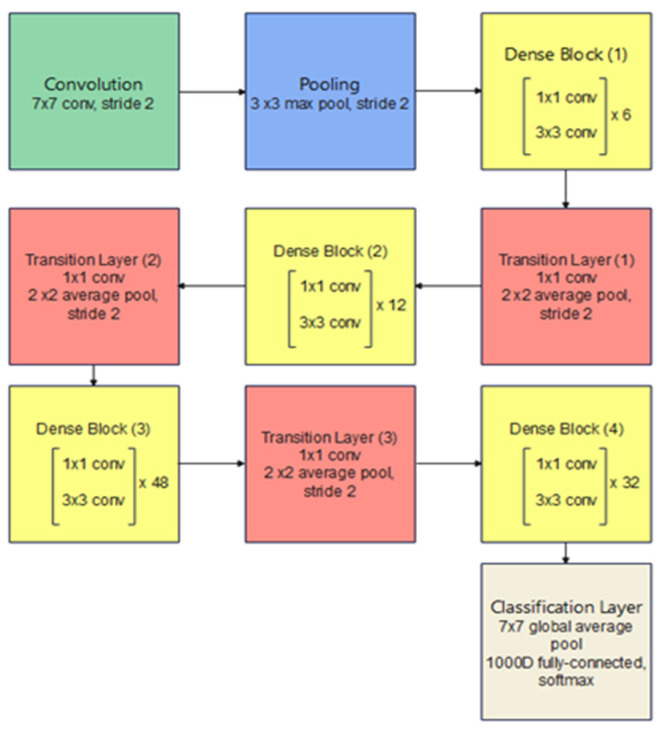
DenseNet-201 architecture, every convolutional layer corresponds to the series BN-ReLU-Conv.

In an ordinary CNN with *n* layers, every layer is connected to the layer after it, which results in *n* connections. However, in a dense convolutional network (DenseNet) the feature maps of a given layer are connected to all consecutive layers and all the previous feature maps from this layer are connected to this given layer, resulting in (*n*(*n* + 1))/2 connections instead. This leads to many benefits including the need for fewer model parameters as compared with ordinary CNNs, enhancing feature propagation and supporting feature reuse. Additionally, it leads to the obtainment of a regularization effect due to the dense connections, which reduce overfitting with smaller training dataset sizes and a good solution to the famous vanishing gradient problem. Hence, the model is easy to be trained. Figure 6 illustrates a dense block with three layers, in which each layer takes all previous feature maps as inputs. It is important to mention that the connected layers should have identical feature-map sizes and the connection is achieved through concatenation instead of summation that is used in residual blocks. Figure 7 presents the architecture of a DenseNet-201 model [19].

### 3.5. Xception

Xception is another model inspired by Inception; however, the inception modules are replaced by depthwise separable convolutions. Unlike regular convolution, which carries out spatial-wise and channel-wise computation in one step, depthwise separable convolution performs this computation in two steps:

1. Depthwise convolution: A spatial convolution process is carried out independently on each input channel solely.
2. Pointwise convolution: This step is performed using a 1 × 1 convolution to project the channels output (output of the depthwise convolution) into a new channel space.

**Figure 8:**
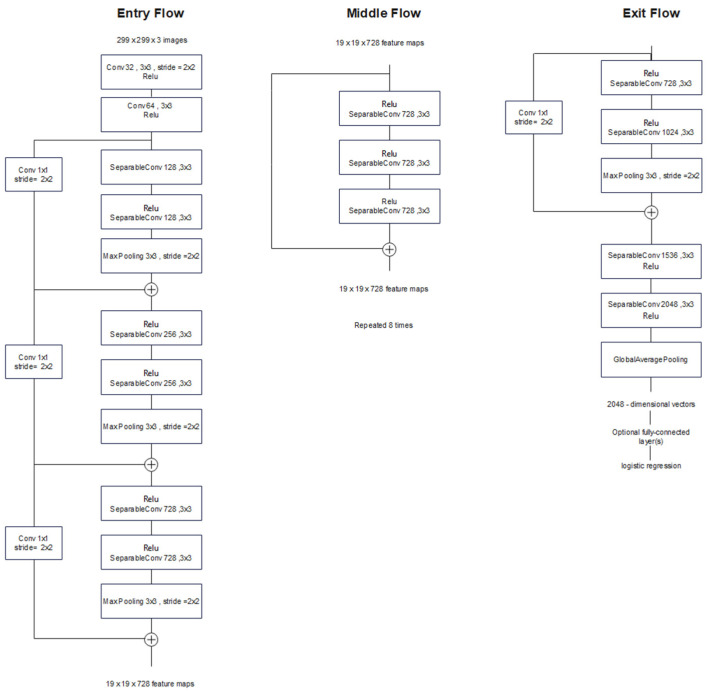
Xception architecture; the input data flow is divided into three parts, starting with the entry flow then moving through the middle flow and ending with the exit flow.

In fact, the Xception model can be considered as a linear stack of depthwise separable convolution layers plus residual connections. Figure 8 demonstrates the architecture of the Xception model. This architecture consists of 36 convolutional layers arranged in 14 modules with residual connections, except for the first and last modules. Every convolution and separable convolution layer is followed by batch normalization (not shown in Figure 8) [20].

## 4. Dataset

One of the most important requirements to build a robust model for the classification purpose is the usage of a large-scale, well-annotated dataset [21]. However, this is a difficult requirement in the medical domain due to the large effort needed for the data-collection and labelling process. The dataset used in this paper is the BreakHis dataset [9]. This dataset contains microscopic biopsy images that are divided into two classes: benign and malignant. These images were collected in the P&D Lab in Brazil. The samples are created from breast tissue biopsy slides stained with Hematoxylin and Eosin (HE). These samples are gathered by surgical open biopsy (SOB) and are annotated by the pathologists of the P&D Lab. Then, digital images are obtained from the breast tissue slides using an Olympus BX-50 system microscope with a relay lens with magnification of 3.3× that is linked to a Samsung digital color camera SCC-131AN. There are 7909 images in this dataset. The images are displayed as magnified images by the magnification factors of 40×, 100×, 200× and 400×, obtained using the conventional lenses of powers 4×, 10×, 20×, and 40×, respectively. Each image is captured as a three-channel (RGB) image with 24-bit color depth and 8 bits per color channel. The image size is 780 × 460 and the format of each image is PNG format. The images are collected from 82 patients with 24 patients diagnosed with benign lesions and the other 58 patients are diagnosed with malignant tumors [9]. Table 2 represents the data distribution in this dataset.

**Figure 9:**
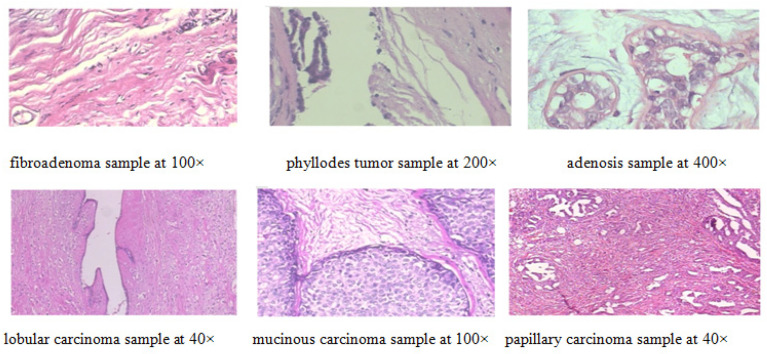
Random samples selected from BreakHis dataset.

The dataset is divided into 5536 images for the training set, 1580 for the validation set, and 793 images for the test set. This division is selected to be as near as possible to the 70% training, 20% validation, and 10% test splitting ratio. For the magnification-independent experiments, all images are considered irrespective of their magnification factors. They are divided into two classes, which are benign and malignant for the binary classification experiment. On the other hand, these images are divided into eight classes, which are adenosis (A), tubular adenoma (TA), phyllodes tumor (PT), fibroadenoma (F), papillary carcinoma (PC), lobular carcinoma (LC), ductal carcinoma (DC) and mucinous carcinoma (MC) for the multi-class classification experiments. Meanwhile, for the magnification-dependent experiments, the images are selected from each magnification factor and then divided into two classes for binary classification. Then, the same images are divided into eight classes for the multi-class classification experiments. Hence, for magnification-dependent experiments, eight datasets are formed in total (four datasets represent each magnification factor for binary classification and another four datasets for the multi-class classification experiments). For the magnification-independent experiments, two datasets are formed (one for the binary classification and one for the multi-class classification). Each dataset formed is divided into a training set, a validation set, and a test set. Moreover, images in BreakHis dataset are randomly shuffled before forming the datasets to break any possible bias. Table 3 illustrates the exact distribution of images in the training, validation, and test datasets. Figure 9 shows a sample of the images from the BreakHis dataset.

## 5. Methodology

The proposed methodology is presented in this section including the data pre-processing stages, transfer learning strategy, hyperparameter configurations, and the adopted performance metrics.

### 5.1. Data Pre-Processing

**Figure 10:**
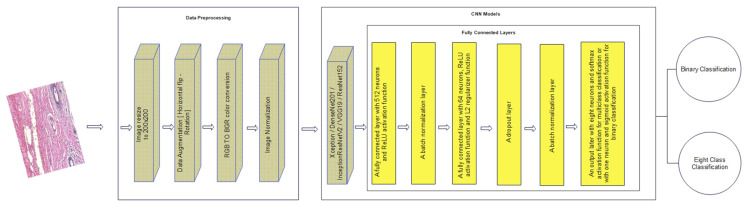
Architecture of the proposed approach including the data pre-processing stage and the designed fully connected layers.

The input images are first resized to 200 × 200 pixels to increase the computational efficiency and reduce the training time. Moreover, since the number of images in the BreakHis dataset is limited, the problem of overfitting can occur [22]. One approach to avoid overfitting is to use data augmentation techniques [23,24]. However, one should carefully select the appropriate data augmentation technique when dealing with medical images. This is attributed to the fact that inappropriate manipulation of essential features can eventually be destructive to the model performance [25]. Only rotation and horizontal flip augmentation techniques are used. Images are rotated randomly with a maximum rotation angle of 180°. The data augmentation used in this study is applied to augment the training input images so that the model never sees the exact same image twice during training process. After the input image is augmented, its color map is changed from RGB to BGR. Then, the input image is normalized. This is mainly needed because when the learning model is fed with input data values that are greatly wide in range, it can make the learning process very difficult, and hence, normalization of the input data is adopted. One method to normalize the input data is to subtract the mean from each data item and divide by the standard deviation. These statistics can be calculated either per the input image or per the dataset [23]. In this paper, the mean and the standard deviation are calculated from the training dataset only, and every input image *x* is then normalized using (1).

(1) $$y=\frac{x-\mu }{\sigma }$$

where *y* is the output image, *μ* is the mean of the training dataset, and *σ* is the standard deviation of the training dataset. Figure 10 indicates the data pre-processing phase.

### 5.2. Transfer Learning Strategy

Since the BreakHis dataset contains a limited number of images, it is not enough to build a high-performance deep learning model through considering only this dataset. It has been proven that the usage of a transfer learning approach for building a deep learning model to predict histopathological images can be much more effective than training the model from scratch using BreakHis dataset [14]. Therefore, in this paper, a transfer-learning approach is used in building a robust deep learning model for the detection of breast cancer in histopathological images. The models that are used in this study are the pre-trained models: Xception, DenseNet201, Inception ResNet V2, VGG19, and ResNet152. These models are pre-trained on the well-known ImageNet dataset that contains more than 14 million natural images. For each model, its classifier is removed (which was trained on the ImageNet dataset) and then our own classifier is used instead. The used transfer learning strategy is divided into two phases:

- Phase 1: For each pre-trained model, all the layers are frozen except for the fully connected layers. Then, training the model is started with a high learning rate on the BreakHis dataset. The earlier layers are frozen, since the model is trained with a small dataset, and hence, there is a high possibility of the model to be overfitted with this high learning rate. It is crucial to avoid the destruction of the already learned features from the ImageNet dataset during this step of training. This phase aims to build the fully connected layers to be able to make predictions on the new problem given the pre-trained base model.
- Phase 2: In this phase, all the layers are unfrozen to fine-tune the model. However, a very low learning rate is adopted; hence, the pre-trained weights are not distorted. Moreover, the weights are updated in a small incremental way to obtain an admirable improvement in terms of classification performance.

### 5.3. Hyperparameters Configurations

It is crucial to have well-designed fully connected layers for the success of a deep learning model [26]. Hence, all the design aspects are considered while building the fully connected layers that are going to be used with each pre-trained model. To fight the well-known problem of overfitting, the dropout and regularization techniques are implemented. For the dropout technique, a dropout probability of 0.5 is adopted. For the regularization technique, L2 regularization is used with L2 regularization factor of 0.001. Moreover, many batch normalization layers are applied in the fully connected layers to stabilize and speed up the training process [21,27,28]. Figure 10 shows the organization of the layers, which can be described as follows:

1. A fully connected layer with 512 neurons. The activation function used in this layer is the ReLU activation function.
2. A batch normalization layer.
3. A fully connected layer with 64 neurons, ReLU activation function and L2 regularizer function.
4. A dropout layer.
5. A batch normalization layer.
6. An output layer with eight neurons and softmax activation function for multi-class classification. For binary classification, it is organized as one neuron and sigmoid activation function.

For the loss functions used, a binary cross entropy loss function is used in case of binary classification. Meanwhile, a categorical cross entropy loss function is used for multi-class classification. Moreover, Adam optimizer is adopted as an optimization algorithm for the learning process with a learning rate of 0.01 for the first phase of the learning process of each model. However, for the second phase of training, each model is trained with two different learning rates, which are 0.00001 and 0.0001, then the best model is selected. This is because the learning rate has the largest influence on the learning process [29]. Hence, to test this influence, these two leaning rates are used as an attempt to generate the best model possible with the highest classification performance. While training each model, the validation accuracy is monitored at the end of each training epoch and if there is any improvement in the validation accuracy, the model weights are saved for that epoch. Hence, at the end of the learning process, the model that has achieved the highest validation accuracy is selected as the output of the training process. Additionally, each training process is carried out for 250 epochs. However, if there is no improvement in the validation accuracy for 60 epochs, the training process is stopped early. These two mechanisms are applied for the two phases of the training process. Finally, a batch size of 32 images is used for the training set.

### 5.4. Performance Metrics

To assess the performance of the studied models, the five most used metrics in the literature are utilized for fair comparison. These metrics are accuracy, precision, recall, F1-score, and Matthew’s correlation coefficient (MCC). These metrics are defined as follows:

(2) $$Accuracy=\frac{TP+TN}{TP+TN+FP+FN}$$

(3) $$Precision=\frac{TP}{TP+FP}$$

(4) $$Recall=\frac{TP}{TP+FN}$$

(5) $$F1\text{-}score=2\times \frac{precision\times recall}{precision+recall}$$

(6) $$MCC=\frac{\left(TP\times TN\right)-\left(FP\times FN\right)}{\sqrt{\left(TP+FP\right)\left(TP+FN\right)\left(TN+FP\right)\left(TN+FN\right)}}$$

where *TP* and *TN* denote the true positive and true negative samples, respectively. In other words, they are the correctly classified malignant and benign samples, respectively. On the other hand, *FP* and *FN* denote the false-positive and false-negative samples, respectively, which describe the incorrectly classified benign and malignant samples. Moreover, the confusion matrix is going to be presented for each model that expresses the different combinations of *TP*, *TN*, *FP*, and *FN* generated by the model. One of the important measures that can assess the classification quality of a deep learning model even if there is an imbalance in the classes of a dataset is *MCC* [30]. Since there is an imbalance in the BreakHis dataset, *MCC* is used to assess the performance of the generated models. *MCC* can be used for binary and multi-class classifications; it is simply a correlation coefficient in the range of −1 to 1. If the value of *MCC* is 1, this indicates a superlative classification, and a value of −1 indicates a total misclassification. On the other hand, an *MCC* value of 0 indicates an average random classification [31]. Additionally, a macro-average technique is used to evaluate the values of precision, recall, and F1-score for each model. Finally, the accuracy is considered as the main metric for this study.

## 6. Experimental Results

In this section, the results for the conducted experiments are going to be presented for each model (magnification-independent binary classification, magnification-independent multi-class classification, magnification-dependent binary classification, and magnification-dependent multi-class classification). These experiments are carried out to test the efficiency of the selected deep learning architectures from all classification aspects. The codes for the experiments are written using Python as a programming language and TensorFlow library version 2.8. The codes are executed through Google Colaboratory environment, which runs entirely on the cloud.

### 6.1. Magnification-Independent Binary Classification

**Figure 11:**
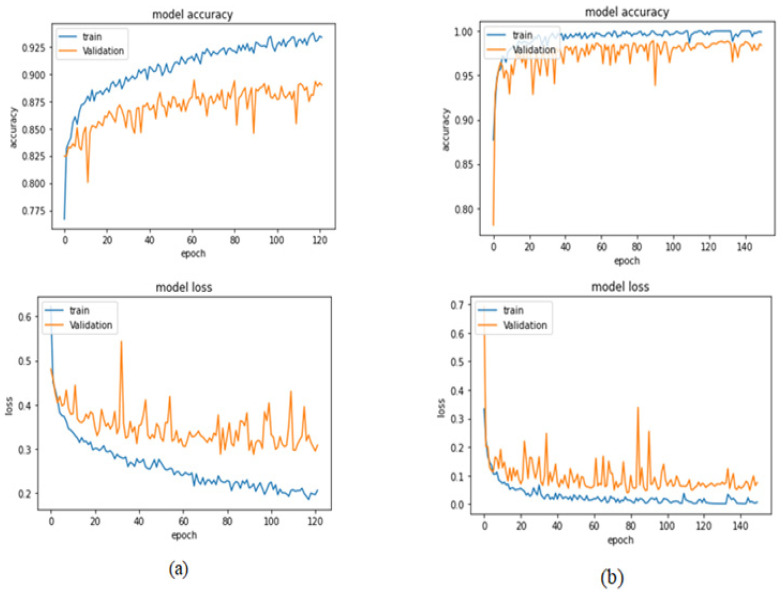
Learning curves for the best obtained results in magnification-independent binary classification, which are achieved by Xception model at 0.0001 learning rate. (**a**) Accuracy and loss curves of Phase 1 of training. (**b**) Accuracy and loss curves of Phase 2 of training.

**Figure 12:**
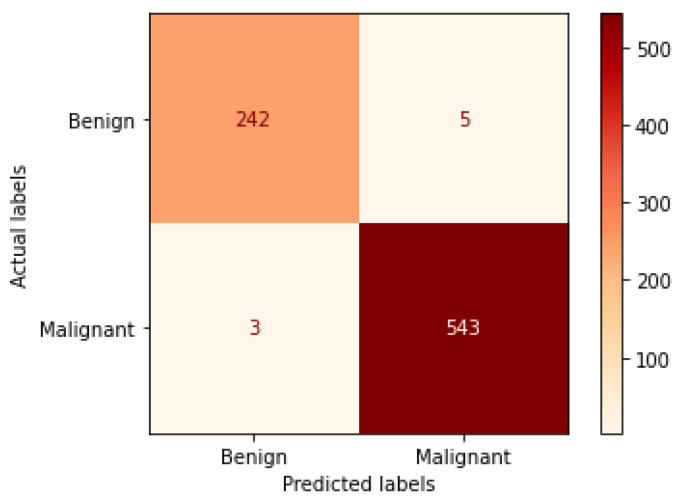
Confusion matrix of Xception model in magnification-independent binary classification experiment.

Table 4 illustrates the performance metrics obtained using the test set for the magnification-independent binary classification experiment. It is clear that the Xception model at a learning rate of 0.0001 achieves the highest results in all of the performance metrics. Moreover, VGG19 at a learning rate of 0.00001 and DenseNet201 at a learning rate of 0.0001 achieve promising results after that for Xception. ResNet152 at a learning rate of 0.0001 achieves the lowest results. However, these results are significantly improved when the learning rate is decreased to 0.00001. Figure 11 and Figure 12 show the learning curves and the confusion matrix of the best model (Xception model). At Phase 1 of training, the best model obtained for Xception achieves a validation accuracy of 89.43% at epoch 62, and the learning process stops early at epoch 122 due to there being no improvement in the validation accuracy for 60 epochs. For Phase 2, the best model obtained achieves a validation accuracy of 98.92% with a 9.49% increase in accuracy as compared to the value obtained in phase 1. This validation accuracy is in synchronization with the test accuracy achieved afterwards (i.e., 98.99%). This indicates that the model is not biased to the data in the validation dataset and the model is fine-tuned correctly. This validation accuracy is obtained at epoch 90 and the training process stops early at epoch 150.

### 6.2. Magnification-Dependent Binary Classification

In this subsection, the magnification-dependent results obtained for the binary classification mode are presented.

#### 6.2.1. 40× Magnification Factor

**Figure 13:**
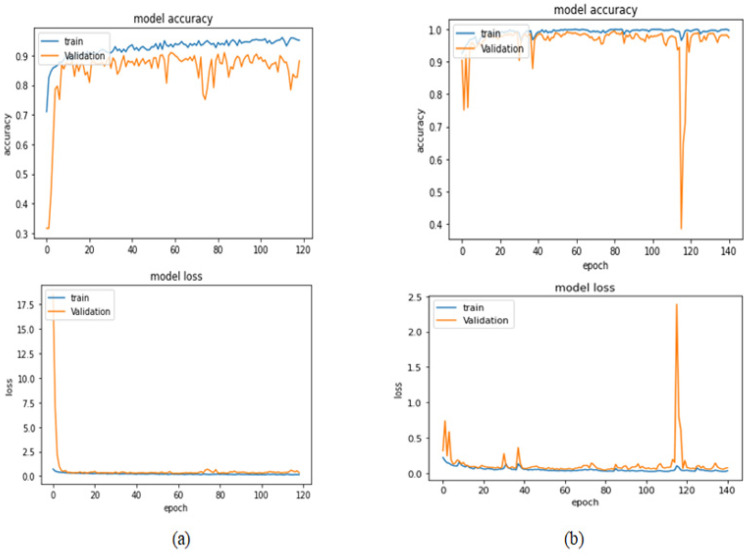
Learning curves for VGG19 at 0.00001 learning rate in 40× magnification dependent binary classification. (**a**) Accuracy and loss curves of Phase 1 of training. (**b**) Accuracy and loss curves of Phase 2 of training.

**Figure 14:**
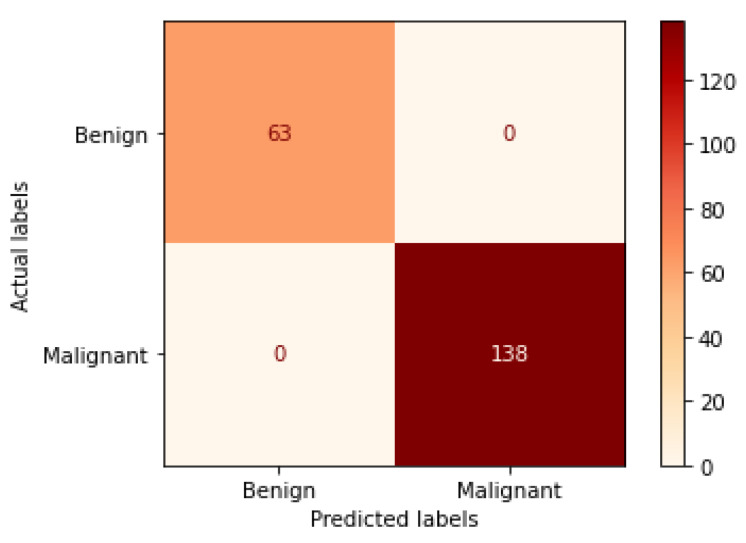
Confusion matrix of VGG19 model in 40× magnification-dependent binary classification.

Table 5 represents the results obtained using the test set for the 40× magnification factor. In this experiment, several models achieve the highest results, which are Xception at a learning rate of 0.0001, DenseNet201 and VGG19 both at a learning rate of 0.00001. The VGG19 model is arbitrarily chosen at a learning rate of 0.00001 to represent its learning curves and confusion matrix as shown in Figure 13 and Figure 14. For Phase 1 of the training process, the best model obtained for VGG19 achieves a validation accuracy of 90.96% at epoch 59 and the learning process stops early at epoch 119 due to there being no improvement in the validation accuracy for 60 epochs. For Phase 2, VGG19 obtains a validation accuracy of 99.50%, which is in synchronization with the test accuracy achieved afterwards (i.e., 100%). This validation accuracy is obtained at epoch 81 and the training process stops early at epoch 141. The least performance is achieved by ResNet152 model at a learning rate of 0.0001.

#### 6.2.2. 100× Magnification Factor

**Figure 15:**
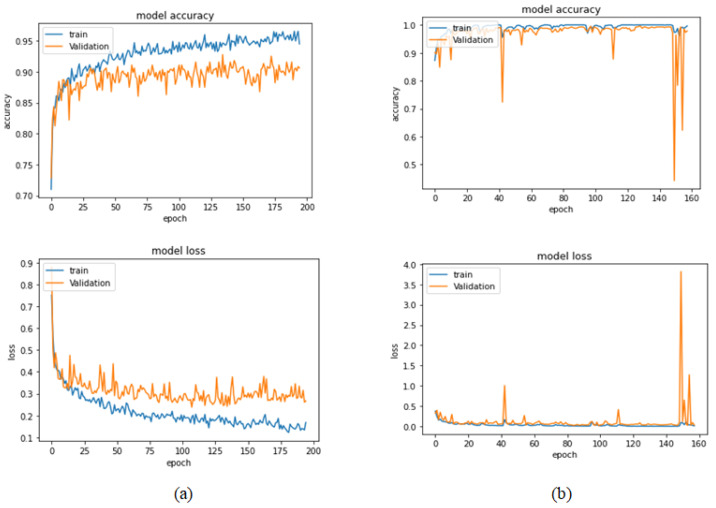
Learning curves for Xception at 0.0001 learning rate in 100× magnification-dependent binary classification. (**a**) Accuracy and loss curves of Phase 1 of training. (**b**) Accuracy and loss curves of Phase 2 of training.

**Figure 16:**
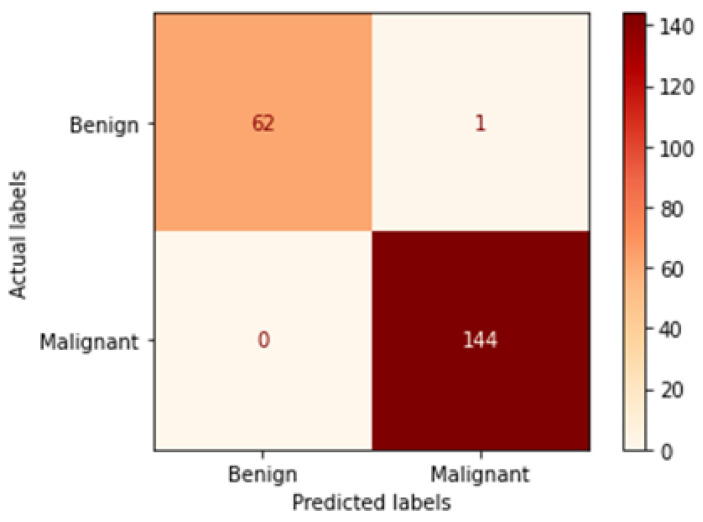
Confusion matrix of Xception model in 100× magnification-dependent binary classification.

Table 6 illustrates the results obtained using the test set for the 100× magnification factor. In this experiment, Xception at a learning rate of 0.0001 and DenseNet201 at a learning rate of 0.00001 performed equally and achieved the highest results. As in the previous magnification factor, ResNet152 at a learning rate of 0.0001 achieves the lowest results. Xception model is chosen arbitrarily to represent its learning curves in Figure 15 and the confusion matrix in Figure 16. For Phase 1 of the training process, the best model obtained for Xception achieves a validation accuracy of 92.79% at epoch 135 and the learning process stops early at epoch 195 due to there being no improvement in the validation accuracy for 60 epochs. For Phase 2, Xception obtains a validation accuracy of 99.52%, which is identical to the test accuracy achieved afterwards (i.e., 99.52%). This validation accuracy is obtained at epoch 98, and the training process stops early at epoch 158.

#### 6.2.3. 200× Magnification Factor

**Figure 17:**
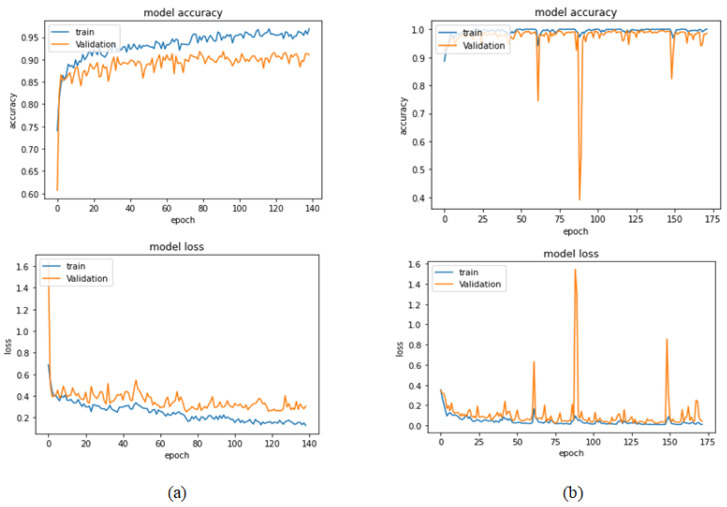
Learning curves for Xception at 0.0001 learning rate in 200× magnification-dependent binary classification. (**a**) Accuracy and loss curves of Phase 1 of training. (**b**) Accuracy and loss curves of Phase 2 of training.

**Figure 18:**
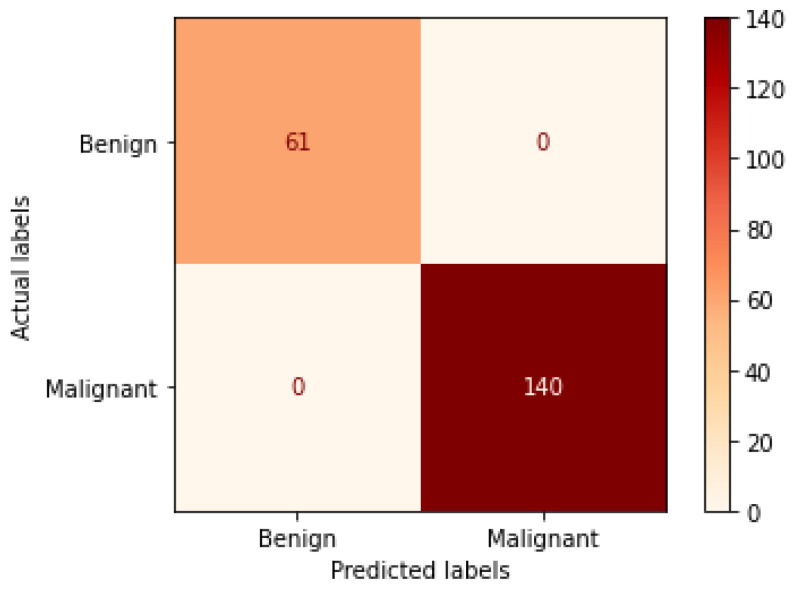
Confusion matrix of Xception model in 200× magnification-dependent binary classification.

Table 7 represents the results obtained using the test set for the 200× magnification factor. In this experiment, Xception at a learning rate of 0.0001 achieves the highest results. In terms of accuracy, DneseNet201 at learning rate of 0.00001 is the second-best model in this experiment. Again, ResNet152 at a learning rate of 0.0001 achieves the lowest results. However, these results dramatically increased at a learning rate of 0.00001. Figure 17 and Figure 18 illustrate the learning curves and confusion matrix for Xception. For Phase 1 of the training process, the best model obtained for Xception achieves a validation accuracy of 91.79% at epoch 79 and the learning process stops early at epoch 139. For Phase 2, the Xception achieves a validation accuracy of 99.75%, which is synchronized with the test accuracy achieved afterwards (i.e., 100%). This validation accuracy is obtained at epoch 112 and the training process stops early at epoch 172.

#### 6.2.4. 400× Magnification Factor

**Figure 19:**
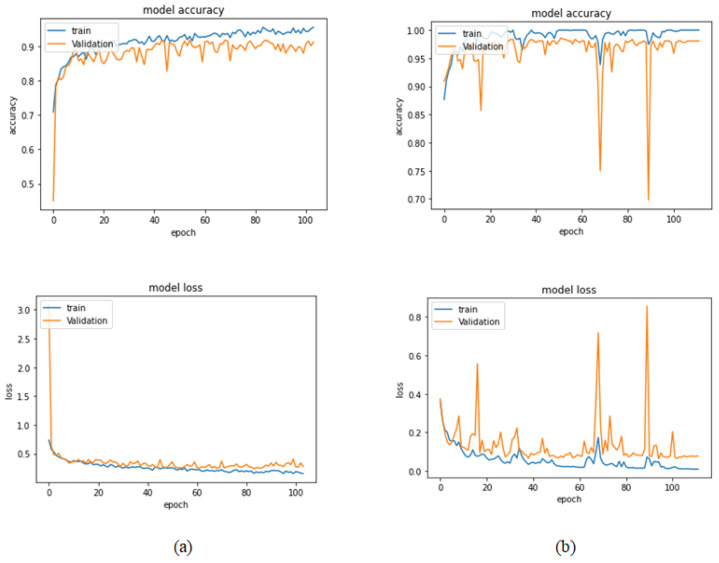
Learning curves for Xception at 0.0001 learning rate in 400× magnification-dependent binary classification. (**a**) Accuracy and loss curves of Phase 1 of training. (**b**) Accuracy and loss curves of Phase 2 of training.

**Figure 20:**
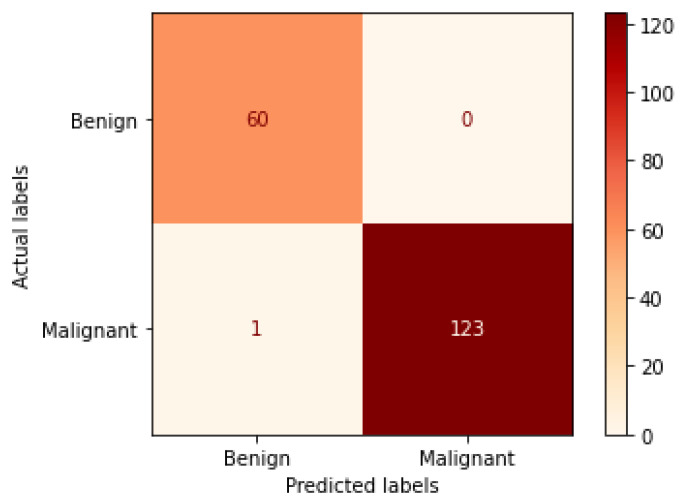
Confusion matrix of Xception model in 400× magnification-dependent binary classification.

Table 8 illustrates the results obtained using the test set for the 400× magnification factor. In this experiment, Xception at a learning rate of 0.0001 achieves the highest results. In terms of accuracy, Inception ResNet V2 at a learning rate of 0.0001 is the second-best model and ResNet152 at a learning rate of 0.0001 achieves the lowest values. Figure 19 and Figure 20 illustrate the learning curves and confusion matrix for Xception. For Phase 1 of the training process, the best model obtained for Xception achieves a validation accuracy of 91.76% at epoch 44 and the learning process stops early at epoch 104. For Phase 2, the Xception achieves a validation accuracy of 98.63%, which is synchronized with the test accuracy achieved afterwards (i.e., 99.46%). This validation accuracy is obtained at epoch 52 and the training process stops early at epoch 112.

### 6.3. Magnification-Independent Multi-Classification

**Figure 21:**
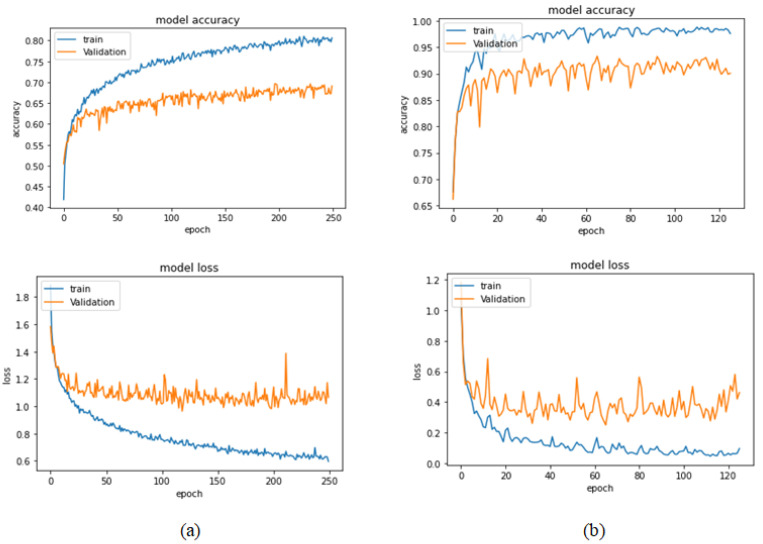
Learning curves for Xception at 0.0001 learning rate in magnification-independent multi-classification. (**a**) Accuracy and loss curves of Phase 1 of training. (**b**) Accuracy and loss curves of Phase 2 of training.

**Figure 22:**
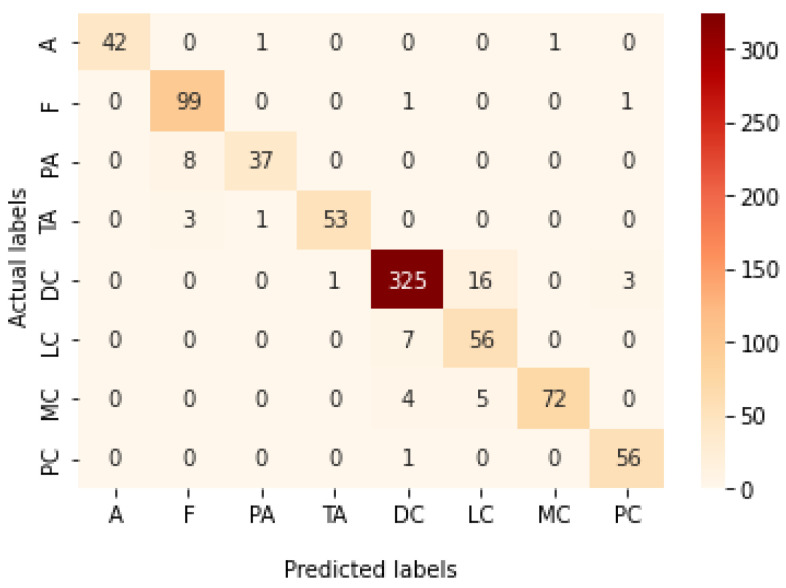
Confusion matrix of Xception model in magnification-independent multi-classification experiment.

Table 9 presents the performance metrics obtained using the test set for the magnification-independent multi-classification mode. The Xception model at a learning rate of 0.0001 achieves the highest results in all the performance metrics. Additionally, DenseNet201 at a learning rate of 0.00001 achieves great results after Xception. Still, ResNet152 at a learning rate of 0.0001 achieves the lowest results. Again, these results are significantly improved when the learning rate is decreased to 0.00001. Figure 21 and Figure 22 present the learning curves and the confusion matrix of the best model for this experiment, which is the Xception model at a learning rate of 0.0001. At Phase 1 of the training process, the best model obtained for Xception achieves a validation accuracy of 69.68% at epoch 197 and the learning process completes the full number of epochs (i.e., 250 epochs) to train the model. For Phase 2, the best model obtained achieves a validation accuracy of 93.29%, which is synchronized with the test accuracy achieved afterwards (i.e., 93.32%). This validation accuracy is obtained at epoch 66, and the training process stops early at epoch 126.

### 6.4. Magnification-Dependent Multi-Classification

In this subsection, the magnification-dependent results obtained for multi-classification mode are presented.

#### 6.4.1. 40× Magnification Factor

**Figure 23:**
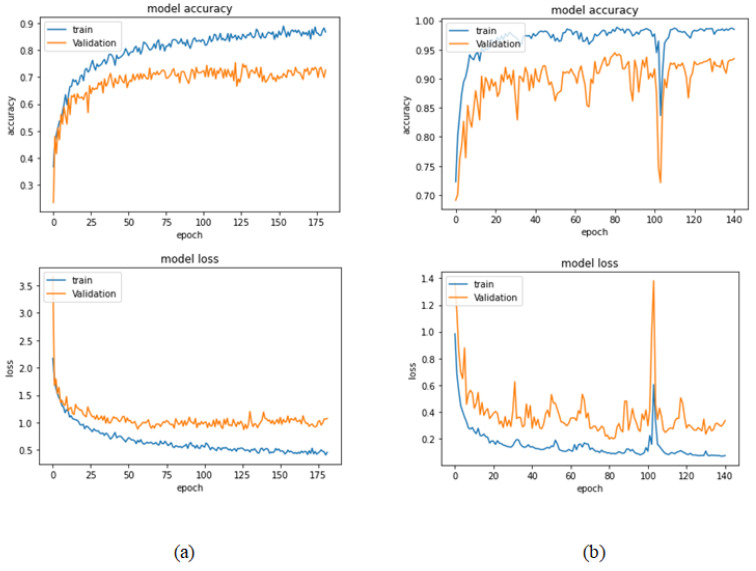
Learning curves for Xception at 0.0001 learning rate in 40× magnification-dependent multi-classification (**a**) Accuracy and loss curves of Phase 1 of training. (**b**) Accuracy and loss curves of Phase 2 of training.

**Figure 24:**
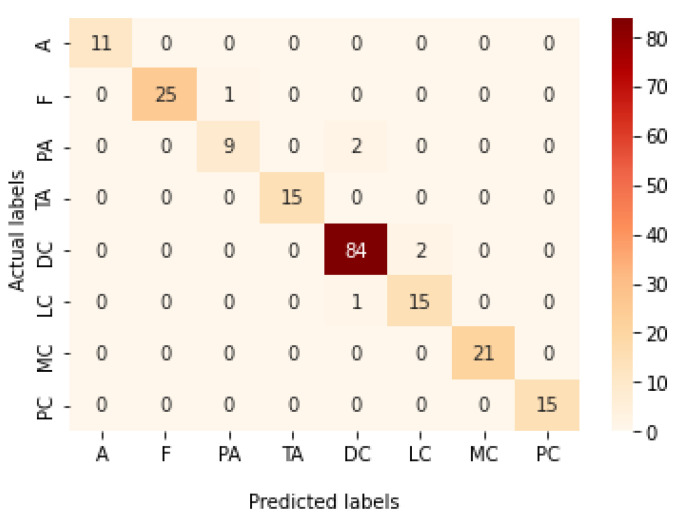
Confusion matrix of Xception model in 40× magnification-dependent multi-classification.

Table 10 represents the results obtained using the test set for the 40× magnification factor. The best model obtained in this experiment is the Xception model at a learning rate of 0.0001. VGG19 also achieves the best results at a learning rate of 0.00001. The least performance is achieved by ResNet152 model at a learning rate of 0.0001. Figure 23 and Figure 24 illustrate the learning curves and confusion matrix for the Xception model. For Phase 1 of the training process, the best model obtained for Xception achieves a validation accuracy of 75.38% at epoch 122 and the learning process stops early at epoch 182 due to there being no improvement in the validation accuracy for 60 epochs. For Phase 2, the Xception model achieves a validation accuracy of 94.47%, which is still in synchronization with the test accuracy achieved afterwards (i.e., 97.01%). This validation accuracy is achieved at epoch 81, and the training process stops early at epoch 141.

#### 6.4.2. 100× Magnification Factor

**Figure 25:**
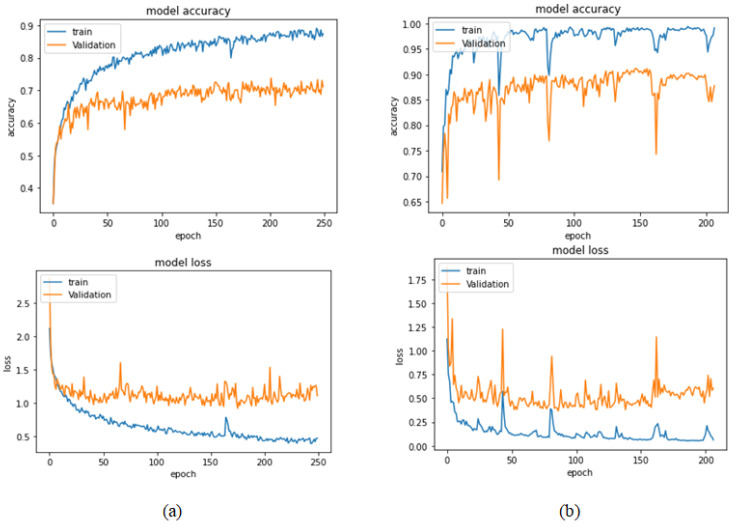
Learning curves for Xception at 0.0001 learning rate in 100× magnification-dependent multi-classification (**a**) Accuracy and loss curves of Phase 1 of training. (**b**) Accuracy and loss curves of Phase 2 of training.

**Figure 26:**
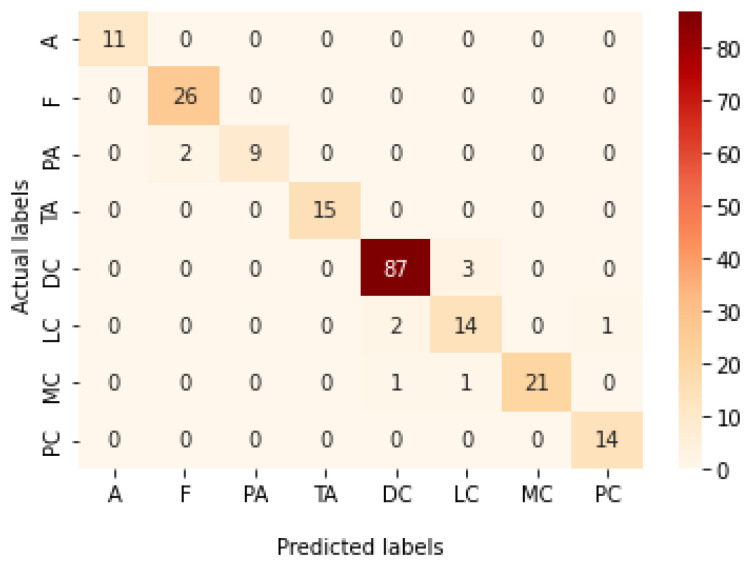
Confusion matrix of Xception model in 100× magnification-dependent multi-classification.

Table 11 illustrates the results obtained using the test set for the 100× magnification factor. In this experiment, Xception at a learning rate of 0.0001 achieves the highest results. Moreover, DenseNet201 at a learning rate of 0.0001 achieves the second-best results. ResNet152 at a learning rate of 0.0001 achieves the lowest results. Figure 25 and Figure 26 illustrate the learning curves and confusion matrix for the Xception model for Phase 1 of the training process. The best model obtained for Xception achieves a validation accuracy of 73.80% at epoch 202, and hence, the training process does not stop early. For Phase 2, Xception achieves a validation accuracy of 91.11%, which is in synchronization with the test accuracy achieved afterwards (i.e., 95.17%). This validation accuracy is obtained at epoch 147, and the training process stops early at epoch 207.

#### 6.4.3. 200× Magnification Factor

**Figure 27:**
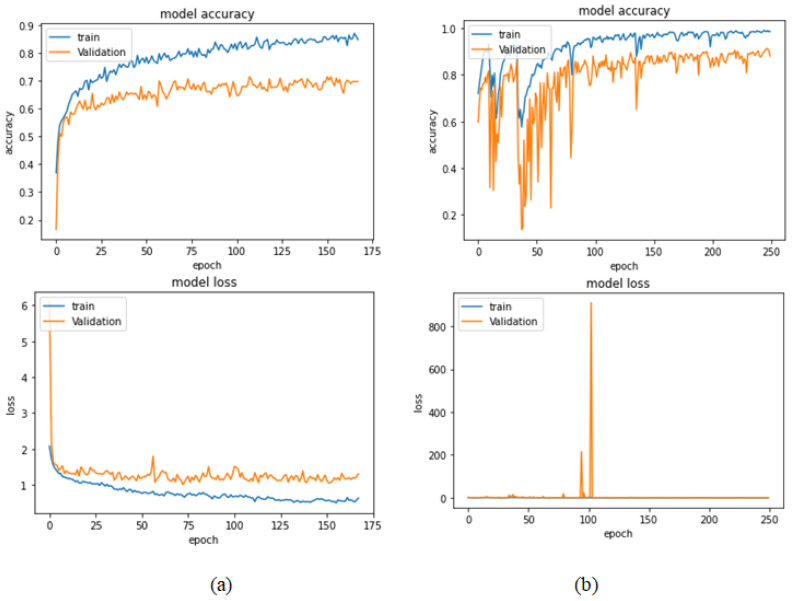
Learning curves for Xception at 0.0001 learning rate in 200× magnification-dependent multi-classification. (**a**) Accuracy and loss curves of Phase 1 of training. (**b**) Accuracy and loss curves of Phase 2 of training.

**Figure 28:**
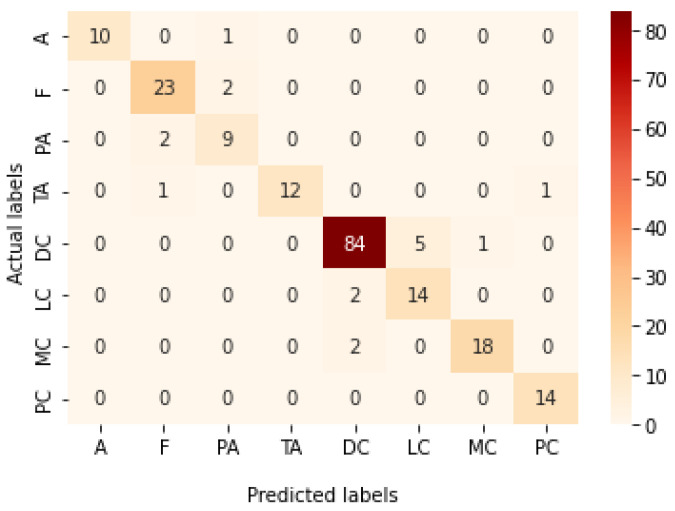
Confusion matrix of Xception model in 200× magnification-dependent multi-classification.

Table 12 represents the results obtained using the test set for the 200× magnification factor. Once again, Xception at a learning rate of 0.0001 achieves the highest results. Inception ResNet V2 at a learning rate of 0.0001 is the second-best model. ResNet152 at a learning rate of 0.0001 achieves the lowest results. Figure 27 and Figure 28 illustrate the learning curves and confusion matrix for Xception. For Phase 1 of the training process, the best model obtained for Xception achieves a validation accuracy of 71.39% at epoch 108 and the learning process stops early at epoch 168. For Phase 2, Xception achieves a validation accuracy of 91.29%, which is synchronized with the test accuracy achieved afterwards (i.e., 91.54%). This validation accuracy is achieved at epoch 247 and the training process completes the total number of epochs.

#### 6.4.4. 400× Magnification Factor

**Figure 29:**
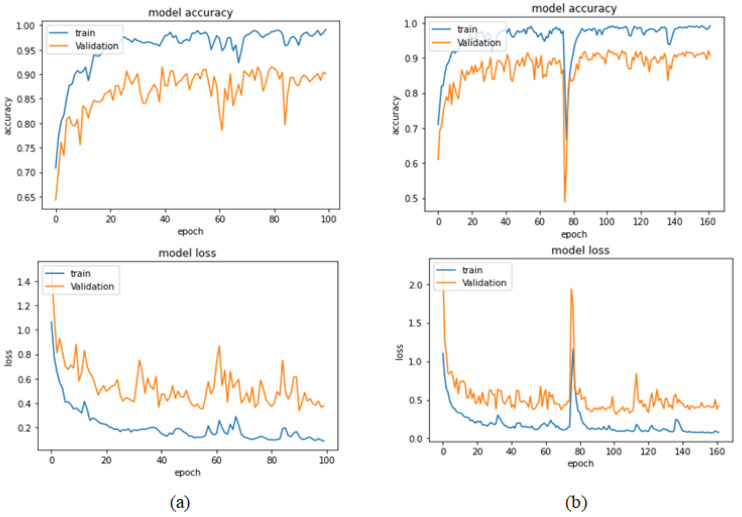
Learning curves for Xception at 0.0001 learning rate in 400× magnification-dependent multi-classification. (**a**) Accuracy and loss curves of Phase 1 of training. (**b**) Accuracy and loss curves of Phase 2 of training.

**Figure 30:**
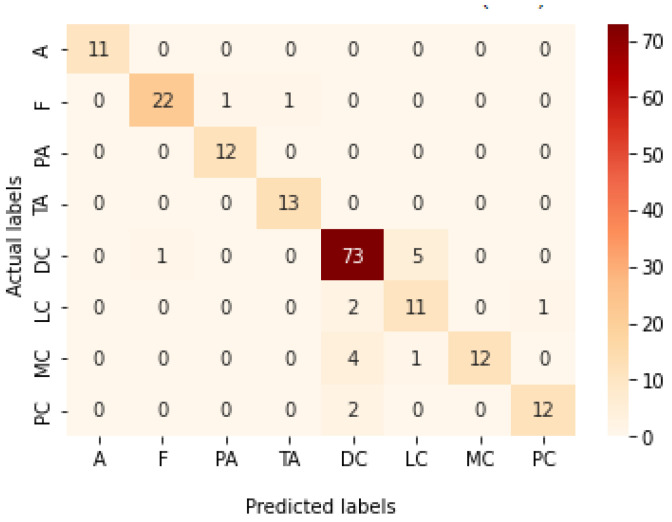
Confusion matrix of Xception model in 400× magnification-dependent multi-classification.

Table 13 represents the results obtained using the test set for the 400× magnification factor. In this experiment, Xception at a learning rate of 0.0001 achieves the highest results in accuracy, recall, F1-score, and MCC metrics. On the other hand, DenseNet201 at a learning rate of 0.0001 achieves a similar value of accuracy but a higher precision value. Once more, ResNet152 at a learning rate of 0.0001 achieves the lowest results. Since the accuracy is the main metric in this study, Xception is considered as the best model for this experiment. Figure 29 and Figure 30 illustrate the learning curves and confusion matrix for Xception. For Phase 1 of the training process, the best model obtained for Xception achieves a validation accuracy of 72.53% at epoch 190, making the learning process finish in epoch 250 (i.e., no early stopping). For Phase 2, Xception achieves a validation accuracy of 92.31%, which is in synchronization with the test accuracy achieved afterwards (i.e., 90.22%). This validation accuracy is achieved at epoch 102, and the training process stops early at epoch 162.

### 6.5. Results of the Best Performing Model

It can be noted from the results of the conducted experiments that the Xception model at a learning rate of 0.0001 achieved the best results in all experiments. These results are summarized in Table 14 for all the conducted experiments.

## 7. Discussion of Results

**Figure 31:**
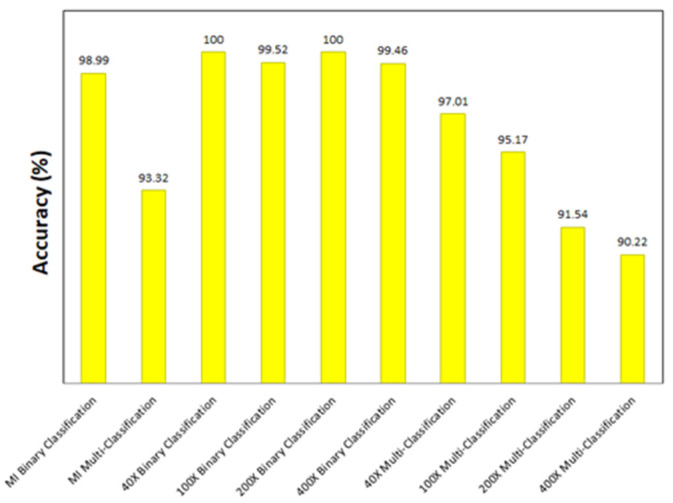
Accuracy achieved by the best model for all the experiments.

It is clear from the results that the Xception model (with a learning rate of 0.0001) achieves the highest classification accuracies in all the experiments. Figure 31 shows the accuracy achieved by the Xception model for each experiment. In addition to Xception, other models perform considerably well. Few examples can be stated in this section to prove this point. In the magnification-independent binary classification experiment, it can be noted that the performance of DenseNet201 at a learning rate of 0.0001 is absolutely comparable to the performance of Xception with a difference of 0.38% in terms of accuracy between the two models. In the same experiment with a learning rate of 0.0001, Inception ResNet V2 is only 0.5% less accurate than Xception. In the 40× binary classification experiment, the comparability of the results is even more evident, in which DenseNet201 and VGG19 at a learning rate of 0.00001 achieve the exact results of Xception at a learning rate of 0.0001. Moreover, the results of 100× binary classification exhibit that DenseNet201 at a learning rate of 0.00001 performs in the same manner as Xception. Thus, the main objectives of the study have been achieved by attaining the most-efficient model as the best performing model in all the experiments and other models that can achieve similar efficacy in specific scenarios.

In order to compare our study with state-of-the-art studies mentioned in the literature, the following points of comparison are used:

- The accuracy of the single best model;
- The number and type of the conduced experiments in the study;
- The usage of various models.

Clearly, our single best performing model outperforms all the approaches in the literature in terms of accuracy. In comparison to Boumaraf et al. [8], Xception model scores higher accuracy than their single proposed model in all the experiments. They conducted the various experiments in a similar manner to our study; however, they did not target the possibility of developing more than one model in the experiments. Our top performing model has achieved higher accuracy values than that achieved by Davoudi et al. [10] in their conducted experiments. The efficacy of their model was tested only in magnification-independent binary classification. They did not produce a variety of successful models. The same conclusion can be obtained from the comparison with Spanhol et al. [11], Xiang et al. [13], and Liu et al. [15], except that Spanhol et al. [11] and Liu et al. [15] perform binary magnification-dependent experiments and Xiang et al. [13] perform binary magnification-independent experiments. Although Bardou et al. [12] have implemented different approaches to classify breast cancer in histopathological images, they have only implemented these approaches in magnification-dependent binary and multi-class classification and score lower accuracy values than the proposed model. The same comparison can be drawn with the study of Shallu et al. [14], except that they have conducted magnification-independent binary classification.

Noticeably, the Xception model exhibits unique performance capabilities that make it the most successful among all the models in all the experiments. Thus, the results can offer hope that the model can be of assistance for pathologists in the future to diagnose the disease.

In order to draw inferences from the results, the models need to use a larger and more balanced dataset. Although the BreakHis dataset contains 7909 images, this is a meager number compared to the gigantic magnitude of breast cancer, which is very prevalent, as there is a scarcity of digital pathological image datasets. The problem lies in the fact that a huge amount of time and effort is needed in labeling and collecting the data [32,33]. Moreover, the dataset was also imbalanced. This draws attention to the fact that a larger and more balanced dataset should be available.

Additionally, it can be noted that the accuracy achieved by Xception model in binary classification mode is higher than that achieved in the multi-classification mode for all the experiments, whether it is a magnification-dependent or magnification-independent experiment. This observation is expected since eight-class classification is more challenging than binary classification.

## 8. Conclusions

Breast cancer is a leading cause of death in women worldwide. The main objective in this study was to investigate the performance of the pre-trained Xception, DenseNet201, Inception ResNet V2, VGG19, and ResNet152 models on the BreakHis dataset. This was mainly to find a reliable deep learning model that can help pathologists in diagnosing breast tumors in histopathological images of any magnification size and of any tumor type. For this objective, multiple experiments were conducted to test all of these models from all the aspects of classifying breast cancer. As a result, magnification-independent binary classification, magnification-independent eight-class classification, magnification-dependent binary classification, and magnification-dependent eight-class classification were carried out for all the pre-trained models at two different learning rates. Xception has performed outstanding results in these experiments. Hence, it can be considered a scaffold on which further studies can be made and provide even more evidence to consider the model as a future tool to aid pathologists in the process of diagnosis. In the future, the trained networks can be further tested using a larger and balanced dataset with different splitting ratios that can be used to ensure robustness. Moreover, the images can be rearranged to enable patient-level performance testing.

## Figures and Tables

**Table 2:** Distribution of data in BreakHis dataset.

	Benign				Malignant				
	A	F	PT	TA	DC	LC	MC	PC	Sum
40×	114	253	109	149	864	156	205	145	1995
100×	113	260	121	150	903	170	222	142	2081
200×	111	264	108	140	896	163	196	135	2013
400×	106	237	115	130	788	137	169	138	1820
Sum	444	1014	453	569	3451	626	792	560	7909

**Table 3:** Distribution of data in the training, validation, and test datasets.

		Benign				Malignant				
		A	F	PT	TA	DC	LC	MC	PC	Sum
Training	40×	80	176	76	104	606	109	143	102	1396
	100×	79	182	86	105	632	119	155	100	1458
	200×	78	186	76	98	627	114	137	94	1410
	400×	74	166	80	91	551	96	118	96	1272
	Sum	311	710	318	398	2416	438	553	392	5536
Validation	40×	23	51	22	30	172	31	41	28	398
	100×	23	52	24	30	181	34	44	28	416
	200×	22	53	21	28	179	33	39	27	402
	400×	21	47	23	26	158	27	34	28	364
	Sum	89	203	90	114	690	125	158	111	1580
Test	40×	11	26	11	15	86	16	21	15	201
	100×	11	26	11	15	90	17	23	14	207
	200×	11	25	11	14	90	16	20	14	201
	400×	11	24	12	13	79	14	17	14	184
	Sum	44	101	45	57	345	63	81	57	793

**Table 4:** Performance metrics collected for magnification-independent binary classification; the highest results are shown in bold.

Learning Rate	Model	Accuracy (%)	Precision (%)	Recall (%)	F1-Score (%)	MCC
0.0001	Xception	**98.99**	**98.93**	**98.71**	**98.82**	**0.9764**
	DenseNet201	98.61	98.33	98.44	98.38	0.9677
	Inception ResNet V2	98.49	98.24	98.24	98.24	0.9647
	VGG19	97.86	97.01	98.11	97.53	0.9511
	ResNet152	68.85	34.43	50.00	40.78	0
0.00001	Xception	98.61	98.44	98.33	98.38	0.9676
	DenseNet201	98.49	98.24	98.24	98.24	0.9647
	Inception ResNet V2	97.23	96.97	96.54	96.75	0.9351
	VGG19	98.74	98.53	98.53	98.53	0.9705
	ResNet152	97.86	97.76	97.22	97.49	0.9498

**Table 5:** Results of 40× magnification factor in binary classification mode; the highest results are shown in bold.

Learning Rate	Model	Accuracy (%)	Precision (%)	Recall (%)	F1-Score (%)	MCC
0.0001	Xception	**100**	**100**	**100**	**100**	**1**
	DenseNet201	99.50	99.22	99.64	99.42	0.9886
	Inception ResNet V2	97.51	96.32	98.19	97.17	0.9449
	VGG19	99.50	99.22	99.64	99.42	0.9886
	ResNet152	81.09	86.09	70.70	73.22	0.5467
0.00001	Xception	99	98.46	99.28	98.85	0.9773
	DenseNet201	**100**	**100**	**100**	**100**	**1**
	Inception ResNet V2	99.50	99.22	99.64	99.42	0.9887
	VGG19	**100**	**100**	**100**	**100**	**1**
	ResNet152	98.01	97.01	98.55	97.73	0.9555

**Table 6:** Results of 100× magnification factor in binary classification mode; the highest results are shown in bold.

Learning Rate	Model	Accuracy (%)	Precision (%)	Recall (%)	F1-Score (%)	MCC
0.0001	Xception	**99.52**	**99.66**	**99.21**	**99.43**	**0.9886**
	DenseNet201	97.58	96.96	97.37	97.16	0.9433
	Inception ResNet V2	96.62	96.20	95.78	95.99	0.9198
	VGG19	98.55	98.98	97.62	98.27	0.9659
	ResNet152	87.44	91.28	79.81	83.09	0.7016
0.00001	Xception	97.58	96.96	97.37	97.16	0.9433
	DenseNet201	**99.52**	**99.66**	**99.21**	**99.43**	**0.9886**
	Inception ResNet V2	96.62	95.83	96.23	96.02	0.9206
	VGG19	99.03	99.32	98.41	98.85	0.9772
	ResNet152	95.17	95.09	93.40	94.19	0.8848

**Table 7:** Results of 200× magnification factor in binary classification mode; the highest results are shown in bold.

Learning Rate	Model	Accuracy (%)	Precision (%)	Recall (%)	F1-Score (%)	MCC
0.0001	Xception	**100**	**100**	**100**	**100**	**1**
	DenseNet201	98.01	98.10	97.18	97.62	0.9528
	Inception ResNet V2	98.01	98.61	96.72	97.60	0.9531
	VGG19	95.52	94.89	94.47	94.68	0.8937
	ResNet152	69.65	34.83	50.00	41.06	0
0.00001	Xception	97.01	96.10	96.93	96.50	0.9303
	DenseNet201	98.51	98.03	98.47	98.24	0.9649
	Inception ResNet V2	98.01	98.10	97.18	97.62	0.9528
	VGG19	96.02	96.69	93.91	95.15	0.9056
	ResNet152	91.04	89.16	89.87	89.50	0.7903

**Table 8:** Results of 400× magnification factor in binary classification mode; the highest results are shown in bold.

Learning Rate	Model	Accuracy (%)	Precision (%)	Recall (%)	F1-Score (%)	MCC
0.0001	Xception	**99.46**	**99.18**	**99.60**	**99.38**	**0.9878**
	DenseNet201	96.74	97.15	95.43	96.22	0.9257
	Inception ResNet V2	97.83	97.95	97.10	97.51	0.9504
	VGG19	96.20	94.78	97.18	95.79	0.9192
	ResNet152	89.67	90.97	85.46	87.48	0.7623
0.00001	Xception	95.65	94.74	95.48	95.10	0.9022
	DenseNet201	97.28	96.41	97.55	96.95	0.9396
	Inception ResNet V2	95.65	94.27	96.34	95.17	0.9059
	VGG19	96.20	95.50	95.89	95.69	0.9139
	ResNet152	91.04	89.16	89.87	89.50	0.7903

**Table 9:** Results of magnification-independent multi-classification; the highest results are shown in bold.

Learning Rate	Model	Accuracy (%)	Precision (%)	Recall (%)	F1-Score (%)	MCC
0.0001	Xception	**93.32**	**92.98**	**92.36**	**92.44**	**0.9129**
	DenseNet201	91.80	91.04	90.10	90.33	0.8923
	Inception ResNet V2	89.79	90.71	85.87	87.75	0.8650
	VGG19	91.05	91.17	0.8880	89.94	0.8816
	ResNet152	43.51	5.440	12.50	7.580	0
0.00001	Xception	90.79	91.69	88.03	89.59	0.8786
	DenseNet201	93.19	92.80	91.61	92.00	0.9107
	Inception ResNet V2	65.70	60.27	52.83	55.68	0.5342
	VGG19	92.18	91.01	90.61	90.71	0.8972
	ResNet152	86.63	86.04	82.93	84.17	0.8229

**Table 10:** Results of 40× magnification factor in multi-classification mode; the highest results are shown in bold.

Learning Rate	Model	Accuracy (%)	Precision (%)	Recall (%)	F1-Score (%)	MCC
0.0001	Xception	**97.01**	**96.85**	**96.17**	**96.47**	**0.9610**
	DenseNet201	94.53	95.18	94.87	94.76	0.9289
	Inception ResNet V2	94.53	93.09	94.71	93.81	0.9290
	VGG19	66.17	38.88	41.87	39.45	0.5471
	ResNet152	42.79	5.35	12.50	7.490	0
0.00001	Xception	92.04	92.75	90.75	91.42	0.8962
	DenseNet201	91.54	92.33	88.91	90.23	0.8891
	Inception ResNet V2	93.03	92.77	92.29	92.44	0.9089
	VGG19	96.52	96.74	95.01	95.77	0.9546
	ResNet152	83.58	83.28	80.15	80.48	0.7847

**Table 11:** Results of 100× magnification factor in multi-classification mode; the highest results are shown in bold.

Learning Rate	Model	Accuracy (%)	Precision (%)	Recall (%)	F1-Score (%)	MCC
0.0001	Xception	**95.17**	**95.08**	**94.02**	**94.37**	**0.9367**
	DenseNet201	92.27	91.95	90.83	90.99	0.8991
	Inception ResNet V2	86.96	87.98	83.19	84.29	0.8284
	VGG19	89.37	92.99	85.29	88.04	0.8600
	ResNet152	43.48	5.430	12.50	7.580	0
0.00001	Xception	84.06	86.09	79.29	81.69	0.7879
	DenseNet201	89.86	91.16	84.74	87.23	0.8658
	Inception ResNet V2	87.92	88.78	84.54	86.25	0.8399
	VGG19	90.34	88.42	90.14	88.69	0.8752
	ResNet152	80.68	81.18	72.78	75.39	0.7425

**Table 12:** Results of 200× magnification factor in multi-classification mode; the highest results are shown in bold.

Learning Rate	Model	Accuracy (%)	Precision (%)	Recall (%)	F1-Score (%)	MCC
0.0001	Xception	**91.54**	**90.08**	**90.16**	**89.91**	**0.8884**
	DenseNet201	85.07	84.38	79.29	79.20	0.8070
	Inception ResNet V2	89.55	88.07	87.90	87.31	0.8638
	VGG19	54.23	26.65	26.93	24.78	0.3374
	ResNet152	44.78	5.60	12.50	7.73	0
0.00001	Xception	82.59	82.59	76.40	78.68	0.7658
	DenseNet201	86.07	87.79	79.69	82.50	0.8127
	Inception ResNet V2	85.07	85.55	80.61	82.26	0.8006
	VGG19	82.59	81.25	77.42	77.86	0.7699
	ResNet152	62.19	47.33	48.65	46.85	0.4954

**Table 13:** Results of 400× magnification factor in multi-classification mode; the highest results are shown in bold.

Learning Rate	Model	Accuracy (%)	Precision (%)	Recall (%)	F1-Score (%)	MCC
0.0001	Xception	**90.22**	90.99	**89.87**	**89.97**	**0.8725**
	DenseNet201	**90.22**	**91.18**	87.22	88.95	0.8714
	Inception ResNet V2	85.87	86.86	81.34	83.60	0.8133
	VGG19	76.63	73.82	65.42	66.81	0.6894
	ResNet152	48.37	11.94	19.52	14.71	0.2267
0.00001	Xception	84.78	85.62	80.14	81.99	0.7995
	DenseNet201	85.87	85.42	80.94	82.87	0.8137
	Inception ResNet V2	78.80	83.68	71.06	75.84	0.7171
	VGG19	84.24	84.44	80.36	81.79	0.7925
	ResNet152	78.26	79.20	68.45	71.68	0.7109

**Table 14:** Results of the best-performing model (Xception model at a learning rate of 0.0001) in all conducted experiments.

Experiment	Accuracy (%)	Precision (%)	Recall (%)	F1-Score (%)	MCC
Magnification-independent binary classification	98.99	98.93	98.71	98.82	0.9764
Magnification-independent multi-classification	93.32	92.98	92.36	92.44	0.9129
40× binary classification	100	100	100	100	1
100× binary classification	99.52	99.66	99.21	99.43	0.9886
200× binary classification	100	100	100	100	1
400× binary classification	99.46	99.18	99.60	99.38	0.9878
40× multi-classification	97.01	96.85	96.17	96.47	0.9610
100× multi-classification	95.17	95.08	94.02	94.37	0.9367
200× multi- classification	91.54	90.08	90.16	89.91	0.8884
400× multi- classification	90.22	90.99	89.87	89.97	0.8725

## Data Availability

Not applicable.
